# Audit of Documentation of Lifestyle Medicine Factors in the Leicestershire Early Intervention Psychosis Team Clinic Letters

**DOI:** 10.1192/bjo.2024.551

**Published:** 2024-08-01

**Authors:** Tamara Chithiramohan, Shiraz Ahmed, Dafni Gavotsi, Debasis Das

**Affiliations:** Leicestershire Partnership NHS Trust, Leicester, United Kingdom

## Abstract

**Aims:**

Lifestyle medicine promotes the use of therapeutic lifestyle interventions to modify disturbed lifestyle factors which are thought to underlie chronic illnesses, including mental health conditions. It is important to identify and manage any disruptions in factors that lifestyle medicine has identified as being contributory towards sustaining good health. Aims were to identify the extent to which the early intervention in psychosis (EIP) medical team in Leicestershire are enquiring about the pillars of lifestyle medicine.

**Methods:**

There are 6 pillars of lifestyle medicine, namely exercise, sleep, diet, refraining from toxins, positive social interactions and quality personal time. Motivation has been added as the 7^th^ pillar for this audit. Gold standard would be to adequately explore all pillars at each medical review. Retrospective analysis was done of electronic patient records (SystmOne) for all patients on the EIP team case load, available on 19^th^ May 2023. Information was gathered from the most recent medical review, using a predefined audit extraction tool. Information on each pillar was assessed based on whether it was fully explored, mentioned with some detail, mentioned with no further detail, or not mentioned at all. Data collection was carried out by three members of the team (TC, SA and DG).

**Results:**

495 patients were identified and 459 had information from a latest medical review found on SystmOne. For all domains, “not mentioned” was the leader, ranging from 48.6–70.8%). For all domains, except for refraining from toxins, the second most common finding was “mentioned with no further details”.

**Conclusion:**

Our results suggest EIP medical staff are either not discussing many of the seven pillars of lifestyle medicine with patients, or not documenting them in sufficient detail. Limitations of the study include that it was the most recent medical review being audited and there could have been more detail documentation in previous reviews. Distribution of the findings and recommendations from the audit were shared with the team and an educational poster detailing lifestyle factors was created. The online system is being adapted to include an option to input lifestyle factors. Re-audit should be done in 12 months.

